# Elevations in D-dimer levels in patients with *Plasmodium* infections: a systematic review and meta-analysis

**DOI:** 10.1038/s41598-024-84907-x

**Published:** 2025-01-05

**Authors:** Suriyan Sukati, Kwuntida Uthaisar Kotepui, Frederick Ramirez Masangkay, Ching-Ping Tseng, Aongart Mahittikorn, Nsoh Godwin Anabire, Polrat Wilairatana, Kinley Wangdi, Hideyuki J Majima, Apiporn Thinkhamrop Suwannatrai, Wiyada Kwanhian Klangbud, Wanida Mala, Rujikorn Rattanatham, Manas Kotepui

**Affiliations:** 1 Medical Technology, School of Allied Health Sciences, Walailak University, Tha Sala, Nakhon Si Thammarat, Thailand; 2 Hematology and Transfusion Science Research Center, Walailak University, Tha Sala, Nakhon Si Thammarat, Thailand; 3 Medical Technology Program, Faculty of Science, Nakhon Phanom University, Nakhon Phanom, Thailand; 4 Department of Medical Technology, Faculty of Pharmacy, University of Santo Tomas, Manila, 1008 Philippines; 5 Graduate Institute of Biomedical Science, College of Medicine, Chang Gung University, Taoyuan, Taiwan; 6 Department of Laboratory Medicine, Linkou Branch, Chang Gung Memorial Hospital, Taoyuan, Taiwan; 7 Department of Medical Biotechnology and Laboratory Science, College of Medicine, Chang Gung University, Taoyuan, Taiwan; 8 Department of Protozoology, Faculty of Tropical Medicine, Mahidol University, Bangkok, 10400 Thailand; 9 Department of Biochemistry & Molecular Medicine, School of Medicine, University for Development Studies, Tamale, Ghana; 10 West African Centre for Cell Biology of Infectious Pathogens (WACCBIP), Department of Biochemistry, Cell & Molecular Biology, University of Ghana, Accra, Ghana; 11 Department of Clinical Tropical Medicine, Faculty of Tropical Medicine, Mahidol University, Bangkok, 10400 Thailand; 12 HEAL Global Research Centre, Health Research Institute, Faculty of Health, University of Canberra, Canberra, Australia ACT 2617 Kirinari Street,; 13 National Centre for Epidemiology and Population Health, Australian National University, Canberra, Australia 62 Mills Road, ACT 2601; 14 Department of Parasitology, Faculty of Medicine, Khon Kaen University, Khon Kaen, 40002 Thailand

**Keywords:** D-dimer, Fibrin degradation products, *Plasmodium*, Malaria, Systematic review, Malaria, Biomarkers

## Abstract

D-dimer, a byproduct of cross-linked fibrin degradation, arises during the fibrinolysis process, breaking down blood clots in circulation. This systematic review and meta-analysis aimed to synthesize evidence of D-dimer alteration in people with malaria, including variations in disease severity. The systematic review was registered in PROSPERO with registration number CRD42024528245. Searches were performed in EMBASE, Scopus, MEDLINE, PubMed, Nursing & Allied Health Premium, and Journals@Ovid on March 25, 2024, to identify original studies that reported D-dimer in patients with *Plasmodium* infections. The methodological quality of the included studies was assessed using the Joanna Briggs Institute critical appraisal tools. Thematic synthesis and meta-analysis were carried out to synthesize the findings of the included studies. A total of 24 studies were included in the review out of 1,115 records identified. According to the evaluated studies, patients with *Plasmodium* infections had higher D-dimer levels. A meta-analytic evaluation of D-dimer levels between patients with and without *Plasmodium* infections revealed a significant elevation of D-dimer in patients with infection, with high heterogeneity (SMD = 2.11, 95% CI = 0.59; 3.64, *P* = 0.007, *I²* = 98%, 6 studies, 1,418 participants, random-effects model). However, no significant alterations in D-dimer levels were observed following the comparison between patients with severe and uncomplicated malaria, also with high heterogeneity (SMD = 2.54, 95% CI = -1.60; 6.68, *P* = 0.23, *I²* = 99%, 3 studies, 595 participants). The findings suggested that malaria patients have significantly higher D-dimer levels compared to non-malarial individuals. However, there was no significant difference in D-dimer levels between severe and uncomplicated malaria cases. These results highlight the potential of D-dimer as a biomarker for *Plasmodium* infections, but its clinical utility requires further validation. Future studies should prioritize standardizing D-dimer measurement methods, including assay types, threshold values, and sample types, to ensure consistent and reliable application in clinical settings. Additionally, large, multicentric cohorts are needed to establish robust guidelines for incorporating D-dimer into malaria management practices. Further research should also explore the role of D-dimer in the pathogenesis of *Plasmodium* infections to deepen our understanding of their clinical significance.

**Supplementary Information:**

The online version contains supplementary material available at 10.1038/s41598-024-84907-x.

## Introduction

Malaria remains a significant global health concern affecting residents and travelers, particularly in tropical and subtropical regions^1,2^. The disease is caused by mosquito-borne parasites of the *Plasmodium* genus, including *Plasmodium falciparum *(*P. falciparum*), *Plasmodium vivax *(*P. vivax*), *Plasmodium malariae *(*P. malariae*), *Plasmodium ovale *(*P. ovale*), and *Plasmodium knowlesi *(*P. knowlesi*)^3^. While disease severity varies across these species, *P. falciparum* is notably associated with severe malaria^4^. Malaria symptoms can vary widely, ranging from uncomplicated to severe^5^. In uncomplicated cases, patients may experience symptoms like fever, chills, headache, fatigue, and muscle aches^6^. However, as the disease progresses, symptoms can worsen and lead to more severe clinical manifestations. These may include complications such as severe anemia, jaundice, metabolic acidosis, acute respiratory distress syndrome, acute renal failure, shock, neurological abnormalities, and disseminated intravascular coagulation (DIC), which can lead to both thrombosis and bleeding^7^. These complications, particularly in cases of *P. falciparum*, significantly increase mortality, especially in populations with limited access to healthcare, underscoring the importance of biomarkers that can predict disease progression. In the most severe cases, malaria can result in life-threatening conditions such as cerebral malaria (CM). As a severe consequence of *P. falciparum* infection, CM is associated with a considerable mortality risk. It is characterized by neurological involvement and appears clinically as a widespread encephalopathy with a history of fever lasting two to three days, followed by seizures and loss of consciousness^4–6^.

Given the wide range of malaria symptoms, specific laboratory tests are essential for diagnosing and monitoring disease progression. Hematological tests are valuable for this purpose, providing significant findings associated with infection and disease severity. Typical findings include anemia, resulting from the destruction of infected red blood cells^8^, and thrombocytopenia^9^, which is a reduction in platelet count^10,11^. Moreover, leukocytosis or leukopenia may occur, indicating changes in white blood cell count depending on the stage and severity of the infection^12,13^. To confirm the diagnoses and monitor treatment responses, peripheral blood smears for malarial parasites are crucial^14^. Coagulation profiles by coagulation tests such as prothrombin time (PT), activated partial thromboplastin time (aPTT), and fibrinogen levels are crucial in identifying DIC and providing valuable evidence for assessing disease severity^15^.

D-dimer, a byproduct of cross-linked fibrin degradation, arises during fibrinolysis, breaking down blood clots in the circulation^16^. This process generates various molecular mass products known as fibrin degradation products (FDPs). The smallest of these products is the D-dimer/fragment E (DD/E) complex, comprising two covalently linked D-domains from two fibrin monomers cross-linked by coagulation factor XIIIa^17^. Elevated levels of D-dimer can indicate the presence of thrombotic or fibrinolytic activity in the body, serving as a marker of thrombosis and reflecting the breakdown of fibrin clots. Elevated D-dimer levels are indicative of suspected venous thromboembolism (VTE), such as deep vein thrombosis (DVT) and pulmonary embolism (PE)^18^, as well as DIC^19^. Inflammatory processes in various conditions, such as infection, can stimulate the activation of the coagulation cascade^20,21^. This activation leads to the generation of fibrin clots, which are subsequently broken down through fibrinolysis. As a result of this process, D-dimer is released into the bloodstream. The level of D-dimer can be measured in plasma, serum, or whole blood^22^. Plasma is commonly used for lab testing, while whole blood is preferred for rapid tests^22^. Regarding normal and critical findings, a plasma D-dimer level below 0.50 µg/mL is considered within the normal range. In contrast, a value of 0.50 µg/mL or higher is defined as positive^23^. Elevated D-dimer levels serve as sensitive indicators preceding the onset of DIC’s clinical symptoms^24^. The sensitivity of D-dimer as an early marker of DIC allows clinicians to initiate prompt diagnostic evaluation and therapeutic interventions in individuals suspected of developing DIC, thereby potentially improving patient outcomes by mitigating the progression of this life-threatening condition.

In other infectious conditions, such as the coronavirus disease (COVID-19), D-dimer has been used as a vital coagulation and disease severity marker^25,26^, suggesting that it may also play a significant role in *Plasmodium* infections. A study suggest that the proportion of DIC in malaria infection can vary based on infection severity and comorbidities^27^. Some studies propose that elevated D-dimer levels may be a subsequent manifestation of DIC^28,29^, associated with complicated malaria as well as infected patients^29–33^. However, there is inconsistency among the findings regarding D-dimer levels in malaria observed across various studies. Therefore, this systematic review and meta-analysis aimed to synthesize evidence of D-dimer alteration in people with malaria, as well as variations in disease severity. This analysis aimed to enhance the understanding of malaria progression. It may aid in the early detection and proper management of hypercoagulability, thereby reducing the risk of life-threatening complications associated with malaria infection. Additionally, this study seeks to clarify the potential utility of D-dimer as a clinical marker for assessing malaria severity and guiding treatment decisions.

## Methods

### Registration

This systematic review was conducted following the Preferred Reporting Items for Systematic Reviews and Meta-Analyses (PRISMA) guidelines^34^ and registered with PROSPERO with registration number CRD42024528245.

### Search strategy

A comprehensive literature search was conducted to identify studies examining D-dimer in patients with *Plasmodium* infections between March 15 and March 25, 2024. The search included multiple platforms and databases such as PubMed (which encompasses MEDLINE records), EMBASE, Scopus, Nursing & Allied Health Premium, and Journals@Ovid. Keywords and medical subject headings (MeSH terms) related to D-dimer and malaria were used in various combinations depending on the database to maximize the retrieval of pertinent studies (Table S1). Additionally, screening of reference lists of selected studies and searches on Google Scholar were performed to ensure a thorough collection of relevant studies.

### Inclusion and exclusion criteria

Studies were eligible for inclusion if they reported on D-dimer levels in patients with *Plasmodium* infections, regardless of the *Plasmodium* species, and compared these levels to those in non-malarial individuals. Eligible studies were cohort, case-control, and cross-sectional studies. Review articles, case reports, in vitro studies, animal studies, conference abstracts, correspondences, studies involving experimentally infected volunteers, and studies with data on D-dimer levels that could not be extracted were excluded.

### Study selection and data extraction

After retrieving the search results, duplicates were removed using Endnote software (Version 20, Clarivate Analytics, United Kingdom). The titles and abstracts of the remaining records were screened, and non-relevant studies were excluded. Full-text articles of the remaining studies were then examined to identify those that met the eligibility criteria, and reasons for exclusion were specified. Extracted data included study design, location, *Plasmodium* species, participant demographics, diagnostic methods for malaria, assays used for D-dimer measurement, blood sample types, and key findings related to D-dimer levels. Two reviewers (SS and MK) independently selected and extracted data from the included studies. Discrepancies were resolved through discussion to minimize subjectivity and potential errors in data interpretation.

### Methodological quality assessment

The methodological quality of the included studies was assessed using the Joanna Briggs Institute (JBI) critical appraisal tools based on the study design^35^. The tool for cross-sectional studies evaluated domains such as ‘clear inclusion criteria,’ ‘valid and reliable exposure and outcome measurements,’ ‘identification and handling of confounding factors,’ and ‘appropriate statistical analysis.’ For cohort studies, the domains included ‘clear participant inclusion,’ ‘reliable measurement of exposures and outcomes,’ ‘identification and management of confounding factors,’ ‘ensuring participants are free of the outcome at the start,’ ‘complete follow-up,’ and ‘appropriate statistical methods.’ For case-control studies, the tool examined domains such as ‘clear inclusion criteria,’ ‘proper selection and comparability of cases and controls,’ ‘valid and reliable measurement of exposures and outcomes,’ ‘identification and handling of confounding factors,’ ‘appropriate matching,’ and ‘consistent criteria for identifying cases and controls. Two independent authors (SS and MK) conducted appraisals and assessed methodological quality and risk of bias. Any discrepancies were resolved through discussion, and if consensus could not be reached, a third reviewer (AM) was consulted to make the final decision.

### Data synthesis and analysis

The data synthesis and analysis steps were conducted using the methods used in previous studies^36,37^. A narrative synthesis, thematic synthesis, and meta-analysis using a random-effects model were applied to synthesize the findings of the reviewed studies^38,39^. The thematic synthesis identified three primary themes: alteration of D-dimer levels in patients with *Plasmodium* infections, differences in D-dimer levels between patients with and without *Plasmodium* infections, and differences in D-dimer levels between severe and uncomplicated malaria cases. The standardized mean difference (SMD) and 95% confidence intervals (CI) of individual studies were pooled using a random-effects model to account for heterogeneity among studies^39^. The SMD, rather than the weighted mean difference (WMD), was used to pool the effect estimate due to the different scales of D-dimer measurements reported and utilized in the meta-analysis^40^. Heterogeneity was assessed using the *I²* statistic, where an *I²* value greater than 50% indicates significant heterogeneity^41^. The meta-analysis investigated differences in levels of D-dimer between patients with *Plasmodium* infections and those without infections. Additionally, differences in D-dimer levels between severe and uncomplicated malaria were investigated. Subgroup analyses were conducted to investigate whether the location of the study, study design, *Plasmodium* species, age groups, diagnostic methods for *Plasmodium* infection, assays for D-dimer, and blood sample types impacted D-dimer levels.

### Sensitivity analysis and publication bias

Sensitivity analysis (influential analysis) was performed to assess the stability of the meta-analysis results. This involved omitting one study at a time to determine its impact on the overall effect estimate^42^. A fixed-effect model was also applied to verify whether the estimates from the fixed and random-effects models pointed in the same direction. Due to fewer than 10 studies being included in the present meta-analysis, publication bias was not assessed^43^. All statistical analyses were performed using RStudio (Version: 2024.04.2 + 764)^44^.

## Results

### Search results

**Fig. 1:**
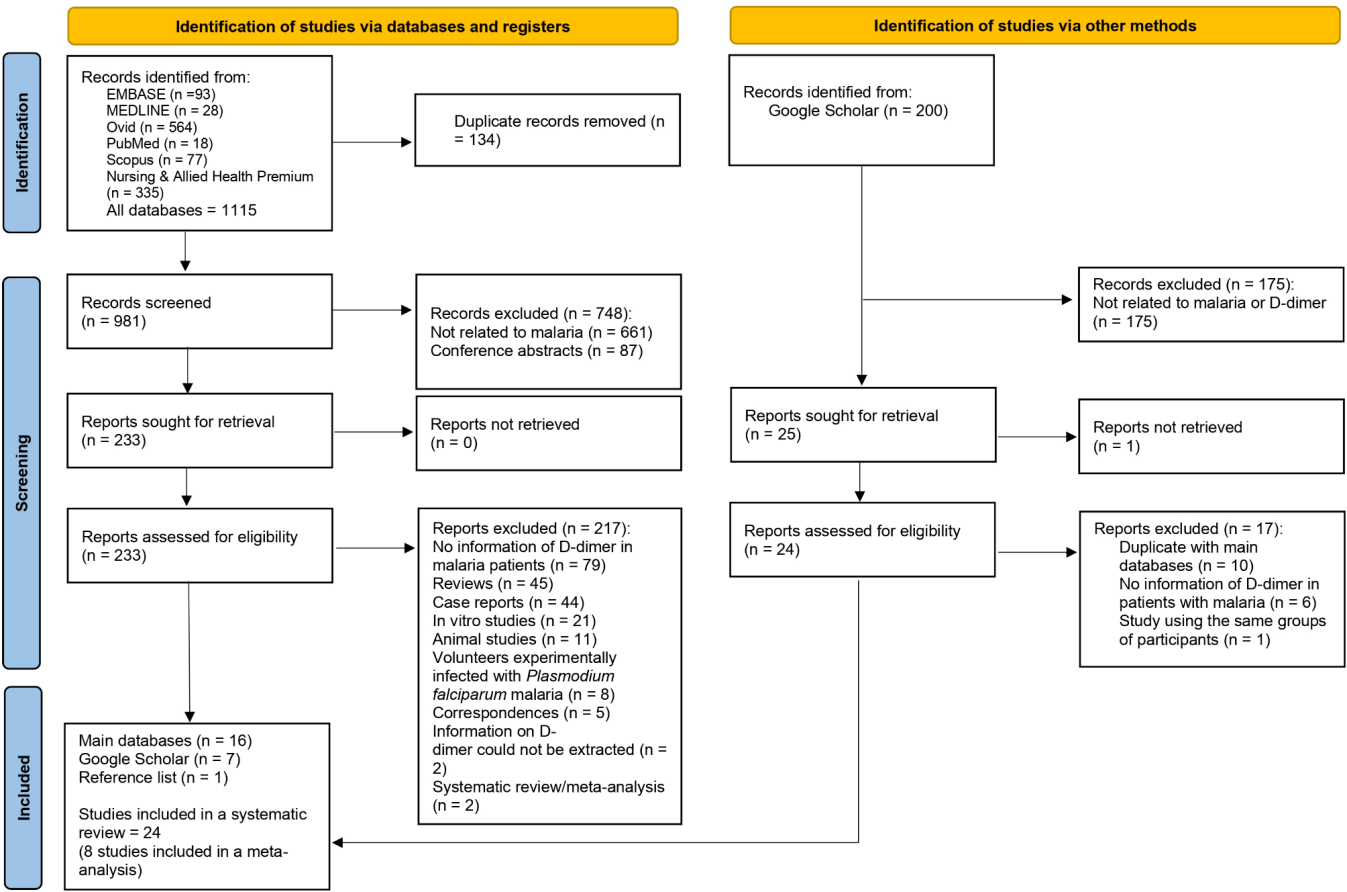
Study flow diagram.

A total of 1115 records were identified from databases. After removing duplicates, 134 records were eliminated, leaving 981 unique records for screening. Of these, 748 records were excluded during the screening phase. Among the exclusions, 661 were unrelated to malaria, and 87 were conference abstracts. The remaining 233 reports were sought for retrieval, and all 233 reports were assessed for eligibility. However, 217 reports were excluded during the eligibility assessment for various reasons. These exclusions included: no information of D-dimer in malaria patients (*n* = 79), reviews (*n* = 45), case reports (*n* = 44), in vitro studies (*n* = 21), animal studies (*n* = 11), studies involving volunteers experimentally infected with *P. falciparum* malaria (*n* = 8), correspondences (*n* = 5), inability to extract information of D-dimer (*n* = 2), and systematic review/meta-analysis (*n* = 2). The reasons for exclusion criteria in detail are demonstrated in Table S1. From the database searches, 16 studies were included^28,30,31,33,45–56^, along with 7 studies from Google Scholar^57–63^ and 1 study from the reference list^64^, resulting in a total of 24 studies that were incorporated into this systematic review^28,30,31,33,45–64^ (Fig. 1).

### Characteristics of included studies

Almost all (95.83%) of the included studies were published between 2010 and 2024, with a majority (62.50%) of the studies published between 2010 and 2019 (Table 1). The 24 studies exhibited diverse characteristics in terms of study design, location, *Plasmodium* species, age groups, diagnostic methods for malaria, assays for D-dimer, and blood sample types (Table 1). Cross-sectional studies were the most common (50.0%), followed by case-control (29.17%) and cohort studies (20.83%). The majority of studies were conducted in Asia (50%), with significant representation from Africa (41.67%) and a smaller portion from Europe (8.33%). *P. falciparum* was the primary species studied (54.17%), with some studies also including non-*P. falciparum* species or not specifying the species. Most studies involved adults (54.17%), although a considerable number also included children (12.50%). Microscopy was the most common diagnostic method for malaria (54.17%), followed by microscopic method/RDT (33.33%). Several immunoassays were utilized for D-dimer measurement, with immunoturbidimetric assay being the most prevalent (20.83%). Blood samples for D-dimer measurement were primarily plasma (58.33%) or serum (25.0%). Details of all studies are demonstrated in Table S2.

**Table 1:** General characteristics of included studies (*n* = 24).

Characteristics	Number of studies (*n*)	%
Publication year		
Before 2000	1	4.17
2000–2009	0	0
2010–2019	15	62.50
2020–2024	8	33.33
Study designs		
Cross-sectional study	12	50.00
Case-control study	7	29.17
Cohort study	5	20.83
Study areas		
Asia	12	50.00
India	9	37.50
Serbia	1	4.17
Pakistan	1	4.17
Israel	1	4.17
Africa	10	41.67
Sudan	4	16.67
Ghana	3	12.50
Kenya	1	4.17
Nigeria	1	4.17
Gabon	1	4.17
Europe	2	8.33
Poland	1	4.17
Germany	1	4.17
*Plasmodium* species		
*P. falciparum*	13	54.17
Non-*P. falciparum*	3	12.50
*P. falciparum*/non-*P. falciparum*	6	25.00
Not specified	2	8.33
Participants		
Adults	13	54.17
Children	3	12.50
Children and adults	5	20.83
Not specified	3	12.50
Methods for detecting *Plasmodium*		
Microscopic method	13	54.17
Microscopic method/RDT	8	33.33
RDT/PCR	1	4.17
Microscopic method/RDT/PCR	1	4.17
Not specified	1	4.17
Assays for D-dimer		
Immunoturbidimetric assay	5	20.83
Enzyme immunoassay	3	12.50
Agglutination assay	3	12.50
Fluorescence immunoassay	2	8.33
Chemiluminescent immunoassay	2	8.33
Immunoassay (type not specified)	1	4.17
Not specified	8	33.33
Blood samples		
Plasma	14	58.33
Serum	6	25.00
Not specified	4	16.67

*PCR* polymerase chain reaction, *RDT* rapid diagnostic test.

### Methodological quality of included studies

The methodological quality of the included studies is shown in Table S3. Twelve cross-sectional studies were included^28,30,31,33,45,48,52,58–61,63^, and exposure was measured validly and reliably in all studies. Most cross-sectional studies (9/12, 75%) provided detailed descriptions of study subjects and settings. Objective and standard criteria were used for measuring the conditions in most cross-sectional studies (10/12, 83.3%). However, the identification and management of confounding factors were less consistent, with only 4 cross-sectional studies identifying confounding factors and addressing them. Outcomes were measured validly and reliably in most cross-sectional studies (9/12, 75%), and appropriate statistical analysis was generally used, although three cross-sectional studies had vague statistical methods.

Seven case-control studies^50,51,54–57,62^ clearly defined the identification criteria for cases and controls. Most case-control studies (5/7, 83.3%) matched cases and controls appropriately. Exposure was measured validly and reliably in all case-control studies. Exposure was measured consistently for both cases and controls across all studies. However, the identification and management of confounding factors were less consistent, with none of the case-control studies fully addressing confounding factors. Outcomes were assessed validly and reliably in most case-control studies (6/7, 85.7%), and appropriate statistical analysis was generally used, although some studies had unclear methods.

Of the five cohort studies^46,47,49,53,64^, the majority (4/5, 80%) had unclear recruitment processes regarding the similarity and population origin of the two groups. The exposure measurement was valid and reliable in all cohort studies. However, the similarity in measurement for assigning people to exposed and unexposed groups was only clear in one study. The identification and management of confounding factors were generally unclear, with only one cohort study not addressing confounding factors at all. Most cohort studies did not provide clear information about whether participants were free of outcomes at the beginning of the studies. Outcomes were assessed validly and reliably in all cohort studies, but the follow-up time was often unclear, with few studies providing sufficient detail on follow-up completeness or strategies to address incomplete follow-up.

### D-dimer levels in patients with *Plasmodium* infections: a qualitative assessment

The reviewed studies reported elevated D-dimer levels in patients with *Plasmodium* infections, with variations observed across different geographical regions and *Plasmodium* species. In Sudan, elevated D-dimer levels were notably observed in Sudanese adults with *P. falciparum* malaria^59^. Similarly, in Gabon, elevated D-dimer levels were also found in Gabonese adults with *P. falciparum* malaria^47^. In India, the results varied significantly. Non-*P. falciparum* malaria cases showed an 8.0% positive D-dimer rate in one study^49^, while another study reported elevated levels in 90.0% of non-*P. falciparum* malaria cases^52^. Contrastingly, Patel et al. found elevated levels of D-dimers in only 1.7% of Indian malaria patients^64^. In a Polish population, all patients exhibited elevated levels of D-dimers^50^. Similarly, a Serbian study noted increased D-dimer levels during the initial phase of malaria^53^. Additionally, Sinha et al. found abnormal D-dimer levels in 80% of non-*P. falciparum* cases^55^.

### Difference in D-dimer levels between patients with *Plasmodium* infections and those without infections: qualitative and quantitative assessments

The eight studies compared D-dimer levels between patients with *Plasmodium* infections and those without infections^31,33,45,54,56–58,63^. Six studies reported a significant elevation of D-dimer levels in patients with *Plasmodium* infections compared to those without infections^31,33,45,56,57,63^. However, two studies presented differing results; Rolling et al. found no significant difference in D-dimer levels between patients with *Plasmodium* infections and uninfected controls^54^; Akwuebu et al. found different results in male and female participants^58^. Gender-specific variations were noted, with Akwuebu et al. observing no difference in D-dimer levels between male patients with uncomplicated malaria and those without malaria, but there was a substantial increase in D-dimer levels in female patients with both complicated and uncomplicated malaria compared to non-malarial patients^58^. The comparison of D-dimer levels between symptomatic and asymptomatic *P. falciparum* infections demonstrated no difference in D-dimer levels^54^.

**Fig. 2:**
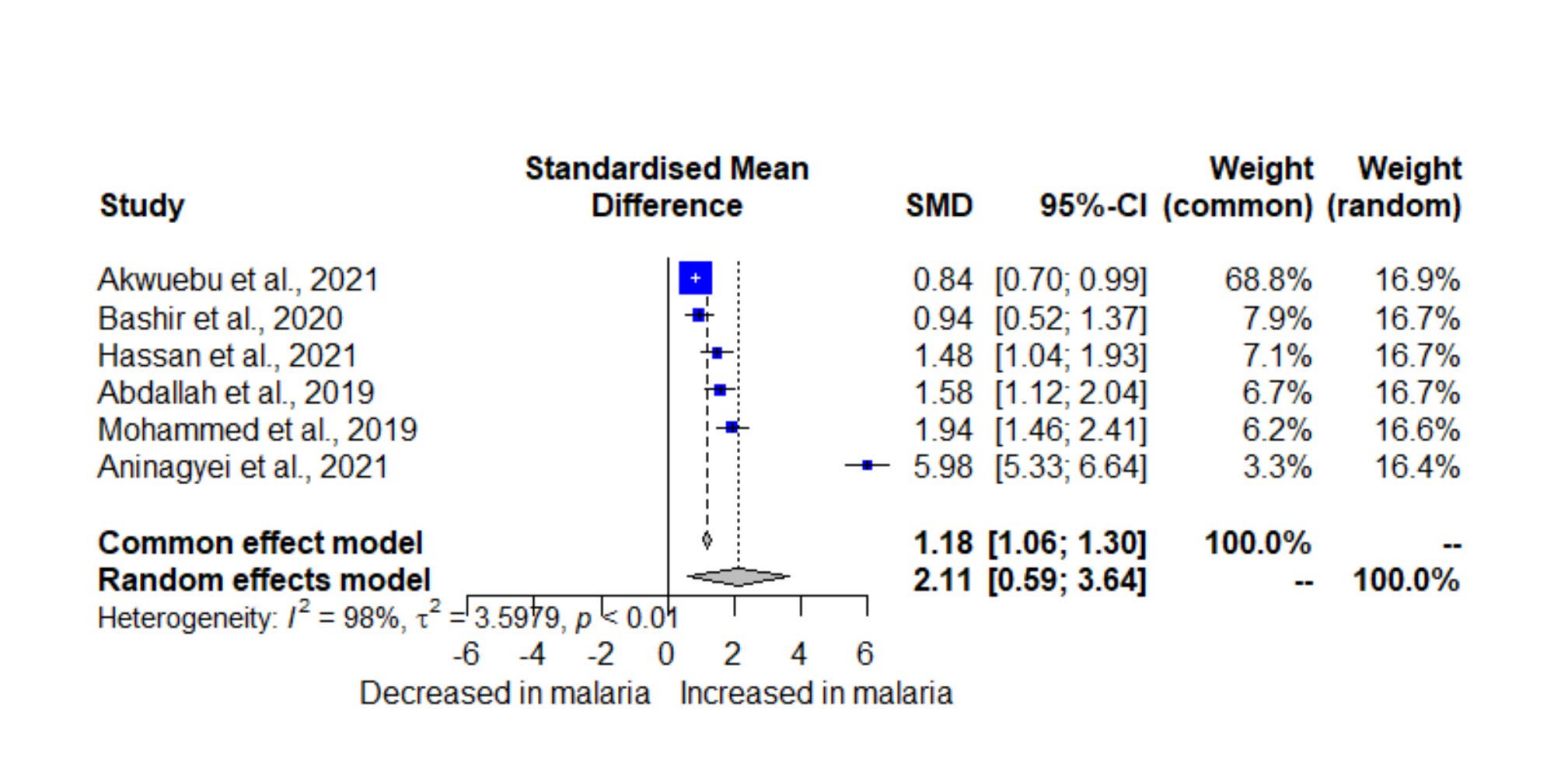
Forest plot displaying significant elevation of D-dimer in patients with *Plasmodium* infections and those without infections. Blue squares, individual study effect estimates; *CI* confidence interval; Gray diamond, the pooled effect estimate; *SMD* standardized mean difference.

The meta-analysis, comprising six studies presenting D-dimer levels in two groups, revealed a statistically significant increase in D-dimer levels in patients with *Plasmodium* infections compared to those without infections (SMD = 2.1148, 95% CI = 0.5856; 3.6440, *P* = 0.0067, *I²* = 97.9%, 6 studies, 1418 participants, random-effects model, Fig. 2). Using the fixed-effect model, the meta-analysis showed a similar direction (SMD = 1.1819, 95% CI = 1.0631; 1.3007, *P* < 0.0001). The subgroup analysis showed significant differences when stratified by country (*P* < 0.01), methods for measuring D-dimer (*P* < 0.01), and blood sample types for D-dimer (*P* < 0.01) (Table 2).

**Table 2:** Subgroup analyses of D-dimer levels between malaria patients and non-malarial individuals.

Subgroup	Test for subgroup differences (random effects model)	SMD (95% CI)	I^2^ (%)	Number of studies
Publication years	0.66			
2020–2024		2.3010 [-0.0978; 4.6997]	98.34	5
2010–2019		1.7510 [ 1.4021; 2.0999]	10.0	
Study design	0.47			
Case-control studies		1.5301 [1.2111; 1.8491]	0	2
Cross-sectional studies		1.1258 [0.9977; 1.2538]	99.0	4
Continent	N/A			
Africa		2.1148 [0.5856; 3.6440]	97.9	6
Country	< 0.01			
Sudan		1.4762 [1.0716; 1.8809]	69.0	4
Nigeria		0.8420 [0.6987; 0.9853]	N/A	1
Ghana		5.9831 [5.3271; 6.6392]	N/A	1
Age ranges	0.24			
Adults		2.5769 [0.3345; 4.8192]	98.0	4
Children		1.1774 [0.4575; 1.8974]	89.0	2
*Plasmodium* species	0.83			
*P. falciparum*		2.1537 [0.2788; 4.0286]	98.0	5
Not specified		1.9360 [1.4580; 2.4139]	N/A	1
Diagnostic method for malaria	0.12			
Microscopy		1.2593 [ 0.7710; 1.7476]	87.0	3
Microscopy/RDT		3.4574 [-1.4802; 8.3950]	99.0	2
Not specified		1.9360 [ 1.4580; 2.4139]	N/A	1
Methods for D-dimer	< 0.01			
Fluorescence immunoassay		3.7749 [-0.5404; 8.0902]	99.0	2
Chemiluminescent immunoassay		1.7008 [1.2576; 2.1440]	46.0	2
Enzyme immunoassay		0.8420 [ 0.6987; 0.9853]	N/A	1
Immunoturbidimetric assay		0.9446 [ 0.5220; 1.3673]	N/A	1
Blood samples for D-dimer	< 0.01			
Plasma		1.3262 [0.9162; 1.7362]	87.0	5
Serum		5.9831 [5.3271; 6.6392]	N/A	1

*RDT* rapid diagnostic test, *CI* confidence interval, *SMD* standardized mean difference, *N/A* not assessed.

### Difference in D-dimer levels between severe and uncomplicated malaria: qualitative and quantitative assessments

Six studies compared D-dimer levels in severe and uncomplicated malaria^28,51,56,58,60,61^ (Table 3). The qualitative assessment highlights that the elevation of D-dimer levels is more pronounced in severe malaria cases^51,56,58,60,61^. Misra et al. provided data from India showing raised D-dimer levels in 53% of falciparum malaria cases with hepatic/renal dysfunction and 30% of cases with uncomplicated falciparum malaria^28^.

**Table 3:** Details of six studies comparing D-dimer levels in severe and uncomplicated malaria cases.

Authors	Study design	Study location	Continent	Year of conduction	Participants for comparison	Plasmodium spp.	Age groups	D-dimer levels in severe and uncomplicated malaria cases
Akwuebu et al.^58^	Cross‒sectional study	Nigeria	Africa	2020	Complicated malaria (25), uncomplicated malaria (426), control (371)	*P. falciparum*	Children	- Female: D-dimer levels were significantly higher in those with complicated malaria compared to those with uncomplicated malaria. - Male: D-dimer levels were significantly higher in those with complicated malaria compared to those with uncomplicated malaria.
Das et al.^60^	Cross‒sectional study	India	Asia	2012	Uncomplicated cases (80), complicated cases (120)	*P. falciparum*	Not specified	The increased plasma D-dimer level was significantly higher in complicated cases compared to uncomplicated cases (40%).
Datta et al.^61^	Cross‒sectional study	India	Asia	Not specified	Complicated cases (32), uncomplicated cases (8)	*P. falciparum*	Adults	D-dimer levels were significantly raised in complicated cases compared to uncomplicated ones.
Meltzer et al.^51^	Retrospective study (case‒control study)	Israel	Asia	2000–2014	Non-immune travelers with malaria (94); - Severe *P. falciparum* cases (10) - Non-severe *P. falciparum* cases (54) - Non-*P. falciparum* cases (30)	*P. falciparum/* non-*P. falciparum*	Adults	D-dimer levels were significantly higher in patients with severe compared to non-severe *P. falciparum* malaria.
Misra et al.^28^	Cross‒sectional study	India	Asia	2008–2009	- Group A: Cases of falciparum malaria with hepatic/renal dysfunction (60) - Group B: Cases of uncomplicated falciparum malaria (20)	*P. falciparum*	All age ranges	- Group A: Raised serum D-dimer (FDP) was found in 53% of cases. - Group B: Raised serum D-dimer (FDP) was found in 30% of cases.
Stauga et al.^32^	Case‒control study	Germany	Europe	2007–2011	Complicated cases (12), Uncomplicated cases (67)	*P. falciparum*	Adults	The median value of D-dimer levels was higher in complicated malaria compared to uncomplicated malaria.

*FDP* fibrin degradation products.

**Fig. 3:**
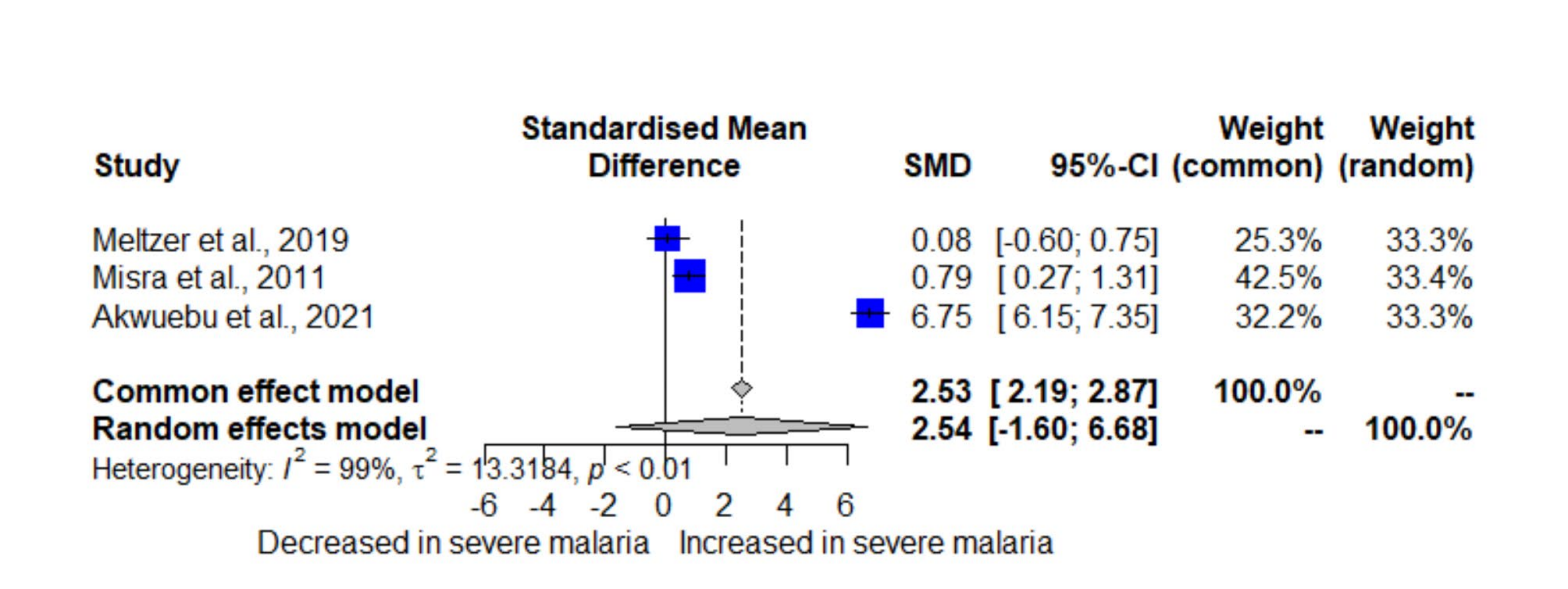
Forest plot displaying significant elevation of D-dimer in severe compared to mild malaria. Blue squares, individual study effect estimates; *CI* confidence interval; Gray diamond, the pooled effect estimate; *SMD* standardized mean difference.

The meta-analysis, which included three studies reporting D-dimer levels in two groups, revealed no significant elevation of D-dimer between severe and uncomplicated malaria (SMD = 2.5406, 95% CI = -1.6036; 6.6848, *P* = 0.2295, *I²* = 99.3%, 3 studies, 595 participants, random-effects model, Fig. 3). Using the fixed-effect model, the meta-analysis showed a different direction (SMD = 2.5301, 95% CI = 2.1904; 2.8698, *P* < 0.0001).

### D-dimer levels in *P. falciparum*, non-*P. Falciparum* infections, and different parasitemia levels: a qualitative assessment

For the elevated D-dimer levels in *P. falciparum* infections, several studies reported significantly higher D-dimer levels in patients with *P. falciparum* malaria compared to those with non-*P. falciparum* infections. Hassan et al. in Sudan found that D-dimer levels were significantly higher in *P. falciparum* malaria compared to non-*P. falciparum* malaria, and particularly D-dimer levels, were elevated in patients with high parasitemia^62^. Similarly, Meltzer et al. in Israel confirmed that D-dimer levels were significantly higher in *P. falciparum* cases, particularly in severe infections^51^.

For the correlation with parasite density, Abdallah et al. in Sudan reported higher D-dimer levels in children with *P. falciparum* malaria, correlating with parasite density^57^. Jabeen et al. in Pakistan observed that D-dimer levels were positively correlated with parasitemia, with notably higher levels in severe *P. falciparum* infections^48^. For the hemorrhagic manifestations in *Plasmodium* infections, Dasgupta et al. in India highlighted the occurrence of high D-dimer levels in patients presenting with hemorrhagic manifestations, regardless of whether they were infected with *P. vivax* or *P. falciparum*^46^.

### Sensitivity analysis

In the meta-analysis comparing D-dimer levels between patients with *Plasmodium* infections and those without, the fixed-effect and random-effects models showed a similar trend, with a statistically significant increase in D-dimer levels in patients with *Plasmodium* infections (Fig. 2). In addition, the influential analysis showed that omitting any single study did not significantly affect the pooled effect estimate (*P* < 0.05, Supplementary File 1). An outlier was identified in the meta-analysis^31^, and after removing this outlier, the results remained stable (SMD = 1.3262, 95% CI: 0.9162; 1.7362, *P* < 0.001, *I*^*2*^: 86.7%, random-effects model, 5 studies, 1418 participants, Supplementary File 1). In the meta-analysis comparing D-dimer levels in patients with severe and mild malaria, the fixed-effect model indicated a different result, showing a statistically significant increase in D-dimer levels in patients with severe malaria compared to those with uncomplicated malaria (Fig. 3). Due to the limited number of included studies, influential analysis, and outlier detection was not performed for the different D-dimer levels between severe and mild malaria.

### Publication bias

Publication bias was not assessed because the number of studies included in the meta-analysis was fewer than 10.

## Discussion

This study highlights a significant association between elevated D-dimer levels and *Plasmodium* infections, particularly with *P. falciparum*. Elevated D-dimer levels, a marker of coagulation activation and fibrinolysis, were observed in a substantial proportion of malaria patients, indicating an underlying hypercoagulable state that may contribute to complications^65^. The findings of the qualitative synthesis reveal that while elevated D-dimer levels are consistently associated with malaria, their prevalence varies across geographic regions and *Plasmodium* species. For example, in Sudanese and Gabonese adults with *P. falciparum* malaria, elevated D-dimer levels were observed in 37.5% and a significant portion of cases, respectively^47,59^. In contrast, studies from India on non-*P. falciparum* malaria cases showed varied results: one reported an 8% positive rate^49^, while another observed elevated levels in 90% of cases^52^. Additionally, Patel et al. reported elevated D-dimers in only 1.7% of Indian malaria patients infected with either *P. falciparum* or non-*P. falciparum* parasites^64^. These geographic and species-specific variations may reflect differences in environmental factors, local health practices, and genetic diversity among human populations and parasites. For instance, in malaria-endemic regions such as Sudan and Gabon, repeated exposure to *P. falciparum* may lead to partial immunity^66^, influencing the host’s inflammatory and coagulation responses. Conversely, in India, the predominance of non-*P. falciparum* species^67^, and potentially lower parasite burden^68^ may result in less pronounced coagulation activation. Additionally, local healthcare practices, including variations in diagnostic methods for malaria and D-dimer, may contribute to discrepancies in D-dimer levels. Notably, methodological variability in measuring D-dimer levels could further amplify these differences. For example, Patel et al. employed an immunoturbidimetric assay^64^, while other studies did not specify their measurement techniques^49,52^. Genetic differences in the host, such as polymorphisms in coagulation-related genes^69,70^ and parasite factors, like strain-specific virulence^71^, may also play a role.

The qualitative synthesis of D-dimer levels between patients with *Plasmodium* infections and those without infections revealed significant insights into the coagulation disturbances associated with malaria. The majority of reviewed studies demonstrate that malaria patients have higher levels of D-dimer compared to non-malarial individuals, highlighting the hypercoagulable state induced by *Plasmodium* infection. This finding across diverse study settings strengthens the evidence that *Plasmodium* infection is associated with significant alterations in coagulation pathways, leading to increased fibrin degradation products such as D-dimer. However, interpreting these findings requires careful consideration of potential limitations in the included studies. One major limitation is the influence of confounding factors, such as concurrent infections, inflammatory conditions, or underlying health issues, which could independently elevate D-dimer levels. For example, co-infections common in malaria-endemic regions, such as bacterial or viral infections^47^, may exacerbate coagulation activation. Additionally, inflammatory conditions^72^, could further confound results. To improve reliability, future studies must rigorously control for confounders to better isolate the effects of *Plasmodium* infection on coagulation pathways and clarify the clinical significance of D-dimer levels in malaria.

The meta-analysis results further substantiate the significant elevation of D-dimer levels in patients with *Plasmodium* infections compared to those without infections. However, the significant heterogeneity observed indicates variability in findings across studies, likely driven by differences in study populations, *Plasmodium* species, the severity of infections, and methodological factors such as D-dimer measurement methods and blood sample types. For instance, variations in D-dimer assays, including immunoturbidimetric, ELISA, or latex agglutination methods, may yield different sensitivity and specificity, leading to discrepancies in reported levels. Similarly, differences in sample types—plasma versus serum—can influence D-dimer measurements due to variations in the presence of clotting factors and sample preparation processes^73^. These findings emphasize the need for standardized protocols for measuring D-dimer, including consistent assay techniques and sample handling procedures for improving the comparability and interpretation of results across studies. Similar to malaria, elevated D-dimer levels have been observed in other diseases, such as COVID-19, and are associated with disease severity and outcomes. For instance, a recent systematic review reported significantly higher D-dimer levels in COVID-19 patients compared to healthy controls^74^. Other reviews have linked elevated D-dimer levels to an increased risk of severe disease and mortality in COVID-19 patients^25,26^. A 3 to 4-fold rise in D-dimer levels has been associated with poor prognosis^75^, underscoring the prognostic value of D-dimer for early identification of patients at risk of severe disease and mortality^76^.

The qualitative synthesis of D-dimer levels between severe and uncomplicated malaria provides crucial insights into the extent of coagulation abnormalities associated with malaria severity. Most reviewed studies indicate a similar pattern where severe malaria cases exhibit significantly higher D-dimer levels compared to non-severe cases, reinforcing the role of D-dimer as a marker of disease severity. This finding is indicative of a more pronounced hypercoagulable state in severe malaria, which aligns with the clinical manifestations of severe disease, including organ dysfunction and higher parasite burdens. Nevertheless, the present meta-analysis did not reveal a statistically significant elevation in D-dimer levels between severe and uncomplicated malaria cases. However, this shortfall of statistical significance should not be interpreted to confute the association between elevated D-dimer levels and malaria severity. Instead, it highlights the limitations inherent in the meta-analysis, including the small number of studies included, substantial heterogeneity, and variability in study populations and methodologies for measuring D-dimer levels. Despite the non-significant result, the high SMD (2.54) with a wide confidence interval (95% CI = -1.60; 6.68) suggests a trend towards higher D-dimer levels in severe malaria cases. This trend aligns with findings from individual studies that report more pronounced coagulation abnormalities in severe cases. Therefore, while the meta-analysis could not confirm a statistically significant association, the observed trend reinforces the need for further research with larger datasets and standardized methodologies to elucidate better the role of D-dimer in differentiating malaria severity.

The qualitative synthesis of the relationship between D-dimer levels, the species of *Plasmodium* infection, and parasitemia levels provides valuable insights into the role of coagulation abnormalities in malaria pathophysiology. The reviewed studies demonstrate that elevated D-dimer levels are associated with *P. falciparum* infections and higher parasitemia^48,57,62^, reinforcing the potential of D-dimer as a marker for malaria severity. The correlation between D-dimer levels and parasitemia was observed in several studies^48,62^, underscoring the impact of parasite load on coagulation processes. Higher parasitemia indicates a more significant burden of infection and a more extensive inflammatory response, which can lead to increased fibrin formation and degradation^77^, thus raising D-dimer levels. Several studies highlight that D-dimer levels are significantly higher in *P. falciparum* malaria compared to non-*P. falciparum* malaria. These findings suggest that *P. falciparum*, known for causing more severe disease, is associated with more significant coagulation disturbances, as reflected by elevated D-dimer levels. Meltzer et al. observed that D-dimer levels were significantly higher in *P. falciparum* compared to non-*P. falciparum* cases^51^ further support the association between this species and pronounced coagulation abnormalities. Nevertheless, non-*P. falciparum* species can also induce significant elevations in D-dimer levels, particularly in cases with hemorrhagic complications, as observed by Dasgupta et al., who reported high D-dimer levels in patients with both *P. vivax* and *P. falciparum* presenting with hemorrhagic manifestations^46^. This underscores the need to carefully monitor coagulation markers in all malaria patients, regardless of the infecting species.

Increased D-dimer levels in *Plasmodium* infection and/or the severity of malaria, as demonstrated by the present systematic review and meta-analysis, align with findings from a previous systematic review on DIC and malaria, which found a higher DIC rate in fatal malaria (82.2%) compared to severe falciparum malaria (14.6%)^27^. Additionally, Sukati et al. demonstrated prolonged or increased prothrombin time (PT) in malaria patients compared to controls, with severe malaria cases showing significantly higher PT than non-severe malaria^15^, indicating coagulation profile abnormalities during *Plasmodium* infection and disease progression. The cause of coagulation alterations, as suggested by a previous review, is that high parasitemia levels may lead to obstruction of hepatic microcirculation, causing abnormalities in the synthesis and secretion of coagulation factors and their inhibitors^78^. Another mechanism involves intravascular fibrin formation and fibrinolysis resulting from plasmin activity in patients infected with *P. falciparum*^77,79^.

The systematic review and meta-analysis have limitations that should be considered when interpreting the findings. First, the meta-analysis was limited in the number of studies, increasing the risk of a Type II error. The qualitative synthesis ensures that the broader spectrum of evidence is adequately represented. Second, high heterogeneity among included studies, as indicated by an *I²* value greater than 50%, may affect the reliability and generalizability of the pooled estimates despite using a random-effects model to account for this heterogeneity. The comparison between the fixed-effect and random-effects models showed that both models indicated elevated D-dimer levels in patients with *Plasmodium* infections compared to those without, suggesting the robustness of this finding despite high heterogeneity across studies. However, the results of the two models differ in the meta-analysis of D-dimer levels between severe and uncomplicated malaria, suggesting that more studies are needed to confirm the robustness of these findings. Additionally, the potential for publication bias cannot be entirely ruled out, especially with few studies. The limited number of included studies prevented a formal assessment of publication bias, raising concerns about the potential impact of unpublished or selectively published studies on the overall findings. Information bias and selection bias are also potential concerns. Selection bias, in particular, may have been introduced by the exclusion criteria, potentially omitting relevant studies that did not report on D-dimer levels in malaria patients. Furthermore, the methodological quality of included studies, assessed using the JBI critical appraisal tools, showed discrepancies that could affect the robustness of the overall conclusions. Inadequate reporting on or control for confounding factors in some studies could also influence the observed associations. Lastly, the inclusion criteria focused on primary research, providing robust quantitative data necessary for reliable comparison and meta-analysis. However, this approach may have limited the breadth of evidence considered. Some valuable insights might have been overlooked by excluding case reports, review articles, in vitro studies, animal studies, conference abstracts, and correspondences. These exclusions were necessary to maintain the integrity and focus of the present systematic review and meta-analysis. Still, they could restrict the generalizability of the findings to broader populations or specific subgroups, potentially limiting the applicability of the results in clinical practice.

The association between elevated D-dimer levels and *Plasmodium* infections, particularly in severe malaria, underscores the critical role of coagulation markers in clinical management. As an indicator of thrombotic activity and fibrinolysis, D-dimer reflects underlying hypercoagulation or DIC, both of which are linked to severe disease outcomes. Its elevation could be an early warning sign of severe complications, enabling clinicians to identify high-risk patients and implement timely and more aggressive management strategies to prevent further progression and complications. Therefore, regular monitoring of D-dimer levels in malaria patients could become a valuable tool in clinical settings, particularly in regions where severe malaria is prevalent. To enhance the clinical applicability of these findings, future research should focus on standardizing D-dimer measurement methods, including assay types, sample handling procedures, and threshold values for clinical interpretation. Conducting multicentric studies across diverse regions with varied healthcare settings and including a broader range of *Plasmodium* species is also critical. Such studies would provide more representative data, accounting for regional and species-specific variations in disease presentation and laboratory findings. Moreover, integrating these studies with longitudinal designs could help determine the prognostic value of D-dimer levels over time, further elucidating their role in predicting disease severity and outcomes. These efforts would strengthen the evidence base and facilitate the integration of D-dimer monitoring into standardized malaria management practices, ultimately improving patient outcomes through early intervention and better resource allocation.

## Conclusion

The findings indicate that malaria patients have significantly higher D-dimer levels compared to non-malarial individuals. However, no significant difference in D-dimer levels was observed between severe and uncomplicated malaria cases. These results highlight the potential of D-dimer as a biomarker for *Plasmodium* infections, but its clinical utility requires further validation. Future studies should prioritize standardizing D-dimer measurement methods, including assay types, threshold values, and sample types, to ensure consistent and reliable application in clinical settings. Additionally, large, multicentric cohorts are needed to establish robust guidelines for incorporating D-dimer into malaria management practices. Further research should also explore the role of D-dimer in the pathogenesis of *Plasmodium* infections to deepen our understanding of their clinical significance.

## Electronic supplementary material

Below is the link to the electronic supplementary material.

Supplementary Material 1

Supplementary Material 2

Supplementary Material 3

## Data Availability

All data relating to the present study are available in this manuscript, Table S1, Table S2, Table S3 files.
