# Supplementary material for: Elevations in D-dimer levels in patients with Plasmodium infections: a systematic review and meta-analysis
Source: Sci Rep. 2025 Jan 5;15:858. doi: 10.1038/s41598-024-84907-x (PMC11701129; doi:10.1038/s41598-024-84907-x)
Supplement: Supplementary file 1 — Supplementary Material 1 [file 41598_2024_84907_MOESM1_ESM.docx]

**Table S1. Search terms**

**General keywords**

“D-dimer fibrin” OR “D-dimer fragments” OR “fibrin fragment D1 dimer” OR “fibrin fragment DD” OR D-dimer OR “fibrin fragment D-dimer” OR “fibrin fragment D”) AND (malaria OR plasmodium OR “Plasmodium Infection“ OR “Remittent Fever“ OR “Marsh Fever“ OR Paludism)

PubMed 15 March 2024

| No. | Key concept | Search terms | Results |
| --- | --- | --- | --- |
| 1. | D-dimer | D-dimer[Text Word] OR D-dimer[MeSH Terms] OR “D-dimer fibrin”[Text Word] OR “D-dimer fragments”[Text Word] OR “fibrin fragment D1 dimer”[Text Word] OR “fibrin fragment DD” [Text Word] OR “D-dimer” [Text Word] OR “fibrin fragment D-dimer”[Text Word] OR “fibrin fragment D”[Text Word] | 18,080 |
| 2. | Malaria | malaria[Text Word] OR malaria[MeSH Terms] OR plasmodium[Text Word] OR “Plasmodium Infection“[Text Word] OR “Remittent Fever“[Text Word] OR “Marsh Fever“[Text Word] OR Paludism[Text Word] | 121,545 |
| 3. | #1 AND #2 | (D-dimer[Text Word] OR D-dimer[MeSH Terms] OR “D-dimer fibrin”[Text Word] OR “D-dimer fragments”[Text Word] OR “fibrin fragment D1 dimer”[Text Word] OR “fibrin fragment DD” [Text Word] OR “D-dimer” [Text Word] OR “fibrin fragment D-dimer”[Text Word] OR “fibrin fragment D”[Text Word]) AND (malaria[Text Word] OR malaria[MeSH Terms] OR plasmodium[Text Word] OR “Plasmodium Infection“[Text Word] OR “Remittent Fever“[Text Word] OR “Marsh Fever“[Text Word] OR Paludism[Text Word]) | 18 |

Embase 15 March 2024

| No. | Key concept | Search terms | Results |
| --- | --- | --- | --- |
| 1. | D-dimer | D-dimer:ti,ab,kw,de OR D-dimer/exp OR D-dimer:ti,ab,kw,de OR “D-dimer fibrin”:ti,ab,kw,de OR “D-dimer fragments”:ti,ab,kw,de OR “fibrin fragment D1 dimer”:ti,ab,kw,de OR “fibrin fragment DD”:ti,ab,kw,de OR “D-dimer”:ti,ab,kw,de OR “fibrin fragment D-dimer”:ti,ab,kw,de OR “fibrin fragment D”:ti,ab,kw,de | 48,531 |
| 2. | Malaria | malaria:ti,ab,kw,de OR plasmodium:ti,ab,kw,de OR ‘Remittent Fever’:ti,ab,kw,de OR ‘Marsh Fever’:ti,ab,kw,de OR Paludism:ti,ab,kw,de OR malaria/exp | 160,874 |
| 3. | #1 AND #2 | (D-dimer:ti,ab,kw,de OR D-dimer/exp OR D-dimer:ti,ab,kw,de OR “D-dimer fibrin”:ti,ab,kw,de OR “D-dimer fragments”:ti,ab,kw,de OR “fibrin fragment D1 dimer”:ti,ab,kw,de OR “fibrin fragment DD”:ti,ab,kw,de OR “D-dimer”:ti,ab,kw,de OR “fibrin fragment D-dimer”:ti,ab,kw,de OR “fibrin fragment D”:ti,ab,kw,de) AND (malaria:ti,ab,kw,de OR plasmodium:ti,ab,kw,de OR ‘Remittent Fever’:ti,ab,kw,de OR ‘Marsh Fever’:ti,ab,kw,de OR Paludism:ti,ab,kw,de OR malaria/exp) | 93 |

Scopus 25 March 2024

| No. | Key concept | Search terms | Results |
| --- | --- | --- | --- |
| 1. | D-dimer | TITLE-ABS-KEY (“D-dimer fibrin” OR “D-dimer fragments” OR “fibrin fragment D1 dimer” OR “fibrin fragment DD” OR D-dimer OR “fibrin fragment D-dimer” OR “fibrin fragment D”) | 38,222 |
| 2. | Malaria | TITLE-ABS-KEY ( malaria OR plasmodium OR "plasmodium infection" OR "remittent fever" OR "marsh fever" OR paludism ) | 162,244 |
| 3. | 1 AND 2 | ( TITLE-ABS-KEY ( ( "D-dimer fibrin" OR "D-dimer fragments" OR "fibrin fragment D1 dimer" OR "fibrin fragment DD" OR d-dimer OR "fibrin fragment D-dimer" OR "fibrin fragment D" ) ) ) AND ( TITLE-ABS-KEY ( malaria OR plasmodium OR "plasmodium infection" OR "remittent fever" OR "marsh fever" OR paludism ) ) | 77 |

Ovid 15 March 2024

| No. | Key concept | Search terms | Results |
| --- | --- | --- | --- |
| 1. | D-dimer AND Malaria | "D-dimer fibrin" OR "D-dimer fragments" OR "fibrin fragment D1 dimer" OR "fibrin fragment DD" OR D-dimer OR "fibrin fragment D-dimer" OR "fibrin fragment D") AND (malaria OR plasmodium OR "Plasmodium Infection" OR "Remittent Fever" OR "Marsh Fever" OR Paludism) {Including Limited Related Terms}  Filter: limit to (ovid full text available and articles with abstracts) | 564 |

MEDLINE 15 March 2024

| No. | Key concept | Search terms | Results |
| --- | --- | --- | --- |
| 1. | D-dimer AND Malaria | (“D-dimer fibrin” OR “D-dimer fragments” OR “fibrin fragment D1 dimer” OR “fibrin fragment DD” OR D-dimer OR “fibrin fragment D-dimer” OR “fibrin fragment D”) AND (malaria OR plasmodium OR “Plasmodium Infection“ OR “Remittent Fever“ OR “Marsh Fever“ OR Paludism) | 28 |

Nursing & Allied Health Premium 24 March 2024

| No. | Key concept | Search terms | Results |
| --- | --- | --- | --- |
| 1. | D-dimer AND Malaria | (“D-dimer fibrin” OR “D-dimer fragments” OR “fibrin fragment D1 dimer” OR “fibrin fragment DD” OR D-dimer OR “fibrin fragment D-dimer” OR “fibrin fragment D”) AND (malaria OR plasmodium OR “Plasmodium Infection“ OR “Remittent Fever“ OR “Marsh Fever“ OR Paludism) | 335 |

Google Scholar 24 March 2024

| No. | Key concept | Search terms | Results |
| --- | --- | --- | --- |
| 1. | D-dimer AND Malaria | (“D-dimer fibrin” OR “D-dimer fragments” OR “fibrin fragment D1 dimer” OR “fibrin fragment DD” OR D-dimer OR “fibrin fragment D-dimer” OR “fibrin fragment D”) AND (malaria OR plasmodium OR “Plasmodium Infection“ OR “Remittent Fever“ OR “Marsh Fever“ OR Paludism) | Screening only the first 200 articles (all records, 6,100) |
